# Dynamical modelling of lipid droplet formation suggests a key function of membrane phospholipids

**DOI:** 10.1111/febs.17238

**Published:** 2024-08-12

**Authors:** Hermann‐Georg Holzhütter

**Affiliations:** ^1^ Institute of Biochemistry Charité – Universitätsmedizin Berlin, Corporate Member of Freie Universität Berlin and Humboldt‐Universität zu Berlin Germany

**Keywords:** computer simulations, dynamic model, lipid droplet, triacylglycerol storage

## Abstract

Cells store triacylglycerol (TAG) within lipid droplets (LDs). A dynamic model describing complete LD formation at the endoplasmic reticulum (ER) membrane does not yet exist. A biochemical‐biophysical model of LD synthesis is proposed. It describes the time‐dependent accumulation of TAG in the ER membrane as the formation of a potential LD (pLD) bounded by spherical caps of the inner and outer monolayers of the membrane. The expansion rate of the pLD depends on the TAG supply, the elastic properties of the ER membrane, and the recruitment of phospholipids (PLs) to the cap‐covering monolayers. Model simulations provided the following insights: (a) Marginal differences in the surface tension of the cap monolayers are sufficient to fully drive the expansion of the pLD towards the cytosol or lumen. (b) Selective reduction of PL supply to the luminal monolayer ensures stable formation of cytosolic LDs, irrespective of variations in the elasto‐mechanical properties of the ER membrane. (c) The rate of TAG supply to the cytosolic monolayer has a major effect on the size and maturation time of LDs but has no significant effect on the TAG export per individual LD. The recruitment of additional PLs to the cap monolayers of pLDs critically controls the budding direction, size, and maturation time of LDs. The ability of cells to acquire additional LD initiation sites appears to be key to coping with acutely high levels of potentially toxic free fatty acids.

## Graphical abstract

Lipid droplets (LDs) are cellular triacylglycerol (TAG)‐storing organelles synthesised at the endoplasmic reticulum (ER). A computational model of LD formation suggests that the uptake rate of additional phospholipids to the LD‐covering leaflets is key in determining the budding direction via cytosol or lumen. The rate of TAG incorporation has a significant impact on the size and maturation time of the LD, but not on the TAG export per individual LD.

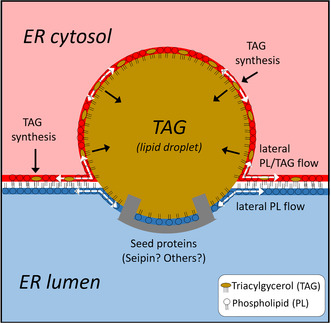

**Abbreviations**
(p)LD(s): (potential) lipid droplet(s)
CPE: cholesteryl palmitate ester
DGAT: diglyceride acyltransferase
ER: endoplasmic reticulum
FA(s): fatty acid(s)
GUV(s): giant unilamellar vesicle(s)
MD: molecular dynamics
PC: phosphatidylcholine
PL(s): phospholipid(s)
TAG: triacylglycerol
VLDL: very low‐density lipoprotein

## Introduction

### What are LDs?

Fatty acids (FAs) are important substrates for the synthesis of cellular membranes and energy (ATP). FAs are very poorly soluble in water and cytotoxic even in low concentrations. Therefore, to stock up on a larger amount of FAs for later use, plant and animal cells esterify them with the sugar derivative glycerol to the neutral lipid triacylglycerol (TAG), which is stored in a special cellular organelle called the lipid droplet (LD). Very similar to lipoproteins, LDs are composed of a hydrophobic lipid core (mostly TAG and sterol esters), covered by a monolayer of phospholipids (PLs), which is decorated with a multitude of specific surface proteins involved in the birth, remodelling, and degradation of the LD [1, 2].

### The ER as the formation site of LDs

It is now widely accepted in the literature that nascent LDs are formed at the membrane of the endoplasmic reticulum (ER) where the enzymes of TAG and PL synthesis are localised. Newly synthesised TAG is initially taken up into the cytosolic monolayer. Its solubility in the PL monolayer is only about 3–5% [3]. If this solubility limit is reached, TAG is released into the inter‐monolayer space where it can accumulate. Molecular dynamics (MD) simulations have shown that model lipid bilayers may sequester TAG (triolein) molecules to the bilayer centre in the form of a disordered, isotropic, mobile neutral lipid aggregate [4]. The existence of spherical TAG lenses embedded in the inter‐leaflet space of bilayers was demonstrated by mixing artificial LDs with giant unilamellar vesicles (GUVs) [5, 6, 7] or by generating TAG lenses in a multilamellar spin‐coated phosphatidylcholine (PC) film [8]. Experiments with such reconstituted systems have provided evidence that an asymmetry in the tension of the outer or inner monolayer, generated, for example, by micropipette aspiration or osmotic stress may shift the TAG lens towards the outer or inner space of the GUV and thus determines the directionality of budding. Tracing the birth of new LDs *in vivo* by fluorescence imaging [9] suggested that individual LDs within one cell acquire TAG at very different rates, pointing to a large variability in the capacity of the underlying molecular processes.

### LD genesis

The current understanding of the molecular details underlying the genesis of LDs at the ER has been outlined in a couple of excellent articles [1, 10, 11, 12, 13, 14]. In brief, *de novo* synthesis of LDs takes place in special ER subdomains, which are defined by the recruitment of several membrane proteins (Fld1, Nem1, Yft2, Pex30 in yeast) that are also involved in the synthesis of TAG and PL. Translocation of TAG into the inter‐monolayer space results in the formation of a lipid lens. The lens forms a spherical bulge towards the cytosol by bending and enlarging the surface of the cytosolic monolayer. The ER protein seipin appears to mediate the conversion of small nascent LDs into larger mature LDs [15]. When the bulging of the cytosolic cap reaches a critical threshold, it detaches from the ER bilayer to form a new LD. It is likely that the presence of specific surface proteins, such as LDAF1 [16], facilitates the final release step. The bending and growth of the cytosolic monolayer into the cytosol requires not only the support of specific surface proteins but also the incorporation of substantial amounts of additional PLs. In *Drosophila* and mouse cells, the need for additional PC to coat the expanding surface during LD expansion is met by the Kennedy pathway [17].

### Mathematical models of LD formation

Several static, elasto‐mechanical models of LD formation have been proposed in the literature [18, 19, 20, 21, 22, 23] using free‐energy minimization methods to describe shape changes of the pLD and the occurrence of energetic instabilities accounting for LD formation. The dynamics of LD formation can be studied by MD simulations (summarised by Kim *et al*. [24]). They provide valuable molecular‐level insight into the formation of LDs but are still confined to very short time intervals of a few microseconds.

### Aim of this work

This work presents, for the first time, a mesoscopic dynamical model of LD formation covering time and length scales far beyond those accessible by current MD simulations [6]. The model incorporates both biochemical and biophysical aspects of LD biosynthesis. The model simulations aimed to quantitatively estimate how the size and formation rate of nascent LDs are influenced by elastic and kinetic parameters of the ER membrane and the rate of TAG supply. The model is also intended to serve as a building block for kinetic metabolic models of cellular lipid metabolism.

## Results

### Mathematical model

**Fig. 1:**
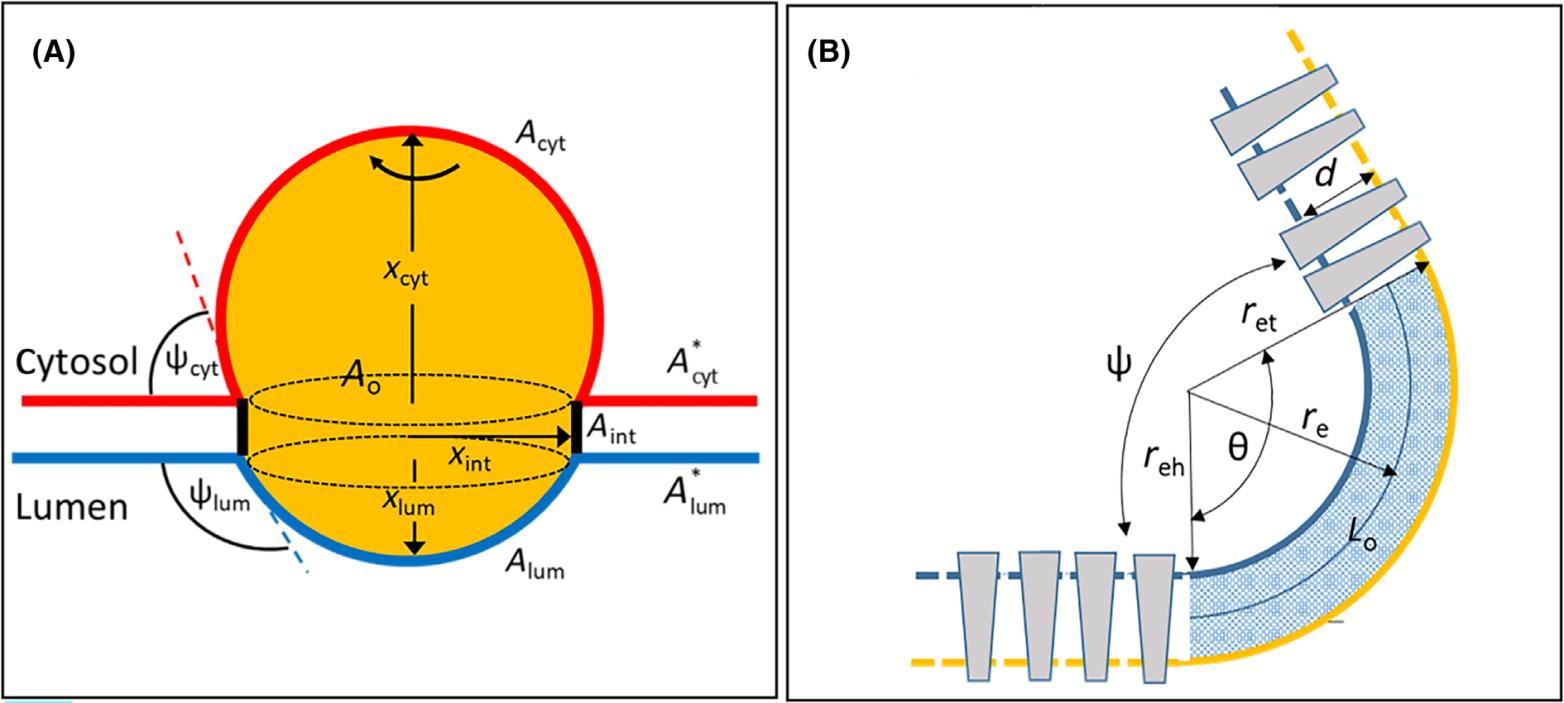
Geometry of the potential lipid droplet (pLD). (A) 2D cross‐section of a spherical pLD. Red and blue lines indicate the cytosolic and luminal monolayer. The black lines mark the front of the lateral inter‐monolayer TAG flow. *A*_cyt_ and *A*_lum_ denote the surface area of the cytosolic and luminal cap. *A*_int_ and the reference area *A*_o_ (indicated by the dotted circles) denote the upper and between‐monolayer surface area of the laterally expanding TAG disk. $A_{\mathrm{cyt}}^{*}$ and $A_{\mathrm{lum}}^{*}$ denote the surface area of the adjacent non‐expanded “reservoir” monolayers, which may exchange PLs with the cap monolayers. The cytosolic and luminal contact angle ψ_cyt_ und ψ_lum_ are enclosed by the bilayer mid‐plane and the dotted tangent lines; they characterise the bending of the respective monolayer in the transition zone (edge) between the cap and reservoir monolayer. (B) The edge between the cap and the adjacent reservoir monolayer is modelled as a sharply curved neck. $r_{\mathrm{eh}}$, $r_{\mathrm{et}}$, $r_{e}$ denote the radii of the head‐group region, tail region, and mid‐plane region of the neck, respectively. The critical elastic length *L*_c_, along which the monolayer is able to relax the thickness mismatch between the thicker neck region (shaded) and the adjacent (white) low‐curvature regions, is approximately given by $L_{c}=\sqrt{\kappa_{b}/T}$ where $\kappa_{b}$ is the bending rigidity and *T* is the line tension [68]. *r*_e_ and *L*_c_ are related by $r_{e}=L_{c}/2/\theta =L_{c}/2/\left(\pi -\psi \right)$.

The accumulation of TAG in the ER membrane is described as an expanding lipid lens (pLD) that is constituted by three compartments: A spherical cytosolic and luminal cap and a circular inter‐monolayer disk. The assumption of spherical caps takes into account that the pLD moves to minimise the energy cost for the unfavourable molecular interactions at the interphase between the oil and aqueous medium [13]. Spontaneous curvature of the bilayer is neglected. The nucleated pLD is treated as incompressible fluid, i.e. absorption of a TAG molecule requires an appropriate expansion of the drop. Expansion of the drop towards the cytosolic and luminal monolayer and along the inter‐monolayer space is described by three coordinates *x*_*i*_ (*i* = cyt, lum, int) (see Fig. 1A).

### The thermodynamic driving force of TAG accumulation in the centre of the ER bilayer

Triacylglycerol is synthesised by enzymes resident on the cytosolic monolayer of the ER membrane. Up to a critical concentration threshold of 3–5% [3], TAG can be accommodated by the monolayer such that the fatty‐acid tails are arranged parallel to the fatty‐acid tails of the PLs, and the oxygen atoms of glycerol backbone are oriented towards the aqueous space [25]. If the TAG content in the monolayer approaches the solubility threshold, excess TAG transits into the hydrophobic inter‐monolayer centre of the ER membrane where it may aggregate and nucleate to an oily lipid lens [9]. Thermodynamically, the net flow of TAG from the monolayer to the bilayer centre occurs if the change of the free energy,

(1) $$\Delta G_{\mathrm{tag}}=\Delta G_{\mathrm{tag}}^{o}+\mathrm{RT}\ln\left(\frac{{\overset{⌢}{\rho }}_{\mathrm{tag}}^{c}}{{\overset{⌢}{\rho }}_{\mathrm{tag}}}\right),$$

is negative. ${\overset{⌢}{\rho }}_{\mathrm{tag}}$ and ${\overset{⌢}{\rho }}_{\mathrm{tag}}^{c}$ denote the (volume) density of TAG in the cytosolic monolayer and the bilayer centre, i.e. the number of molecules per unit volume (= 1 μm^3^). ${\overset{⌢}{\rho }}_{\mathrm{tag}}$ is related to the surface density $\rho_{\mathrm{tag}}$ by ${\overset{⌢}{\rho }}_{\mathrm{tag}}=\rho_{\mathrm{tag}}/d_{m}$ with *d*_m_ being the thickness of the monolayer. For the pLD, we assume a fixed TAG density of ${\overset{⌢}{\rho }}_{\mathrm{tag}}^{c}=6.2\times 10^{8}\;\mu m^{-3}$ (triolein, MW = 885 g·mol^−1^; ${\overset{⌢}{\rho }}_{\mathrm{tag}}^{c}$ = 0.91 g·mol^−1^). The standard free energy change $\Delta G_{\mathrm{tag}}^{o}$ is related to the equilibrium constant $K_{\mathrm{equ}}$ for the exchange of TAG between the monolayer and the pLD:

(2) $$\Delta G_{\mathrm{tag}}^{o}=-\mathrm{RT}\ln\left(K_{\mathrm{equ}}\right).$$

Given a maximal TAG solubility of 5% in the monolayer and 100% in the oil phase of the pLD, then $K_{\mathrm{equ}}=20$ and $\Delta G_{\mathrm{tag}}^{o}$ = −7.43 kJ·mol^−1^. Neglecting entropic changes, the change of the free energy can be equated with mechanical work that is done when moving 1 mol (=*n*_o_ = 6.022 × 10^23^) TAG molecules from the surface of the monolayer to the bilayer centre. We put

(3) $$\Delta G_{\mathrm{tag}}=-F_{\mathrm{tag}}d_{m},$$

where the thickness *d*_m_ of the monolayer is taken as a proxy for the displacement along which the mechanical work is carried out. The force per single TAG molecule is $f_{\mathrm{tag}}=\frac{F_{\mathrm{tag}}}{n_{o}}=-\frac{\Delta G_{\mathrm{tag}}}{d_{m}n_{o}}$. The number of TAG molecules in the monolayer with surface area *A* is $n_{\mathrm{tag}}=A\rho_{\mathrm{tag}}$, where $\rho_{\mathrm{tag}}$ represents the surface density of TAG. Hence, the TAG density gradient across the interface between TAG in the monolayer and the pLD generates a pressure

(4) $$p_{\mathrm{tag}}=n_{\mathrm{tag}}\frac{f_{\mathrm{tag}}}{A}=-\rho_{\mathrm{tag}}\frac{\mathrm{RT}}{d_{m}n_{o}}\ln\left(\frac{d_{m}{\overset{⌢}{\rho }}_{\mathrm{tag}}^{c}}{K_{\mathrm{equ}}\rho_{\mathrm{tag}}}\right).$$

We shall refer to this pressure as the solvation pressure because ultimately it is the increased solubility of TAG in the pLD compared to the monolayer that leads to the accumulation of TAG in the pLD. *p*_tag_ is directed towards the centre of the pLD if $\Delta G_{\mathrm{tag}}<0$, which requires $\rho_{\mathrm{tag}}^{m}>\frac{d_{m}{\overset{⌢}{\rho }}_{\mathrm{tag}}^{c}}{K_{\mathrm{tag}}^{\mathrm{equ}}}=6\times 10^{4}\mu m^{-2}$ if *d*_m_ = 2 nm is taken as monolayer thickness. On the other hand, the membranous TAG density is restricted to an upper limit $\rho_{\mathrm{tag}}^{\#}=1\times 10^{5}\;\mu m^{-2}$, given a maximal TAG solubility of 5% and a PL density of $\rho_{\mathrm{pl}}^{m}\;\approx \;2\times 10^{6}\mu m^{-2}$. Hence, within the narrow TAG density range of $6\times 10^{4}\mu m^{-2}<=\rho_{\mathrm{tag}}^{m}<=1\times 10^{5}\mu m^{-2}$, an inward‐directed solvation pressure between 0 and about 100 kPa can be generated.

### Growth of the oil drop‐driven filling with TAG

According to Pascal's law, the pressure applied to an enclosed fluid will be transmitted without a change in magnitude to every point of the fluid and to the walls of the container. Hence, the solvation pressure *p*_tag_ defined by the Relations (2, 3) drives the expansion of the pLD along the principal coordinates defined earlier. The expansion of the cap surfaces *A*_*i*_ (*i* = cyt, lum) is driven by two processes: (a) equilibration of the difference between the interface pressures *p*_*i*_ and the pressure exerted by membranous TAG and (b) equilibration of the pressures *p*_*i*_ (*i* = cyt, lum, int) among each other. For an incompressible fluid, the latter process occurs at constant volume in terms of an iso‐volumetric drift, which aims to equalise the pressures *p*_*i*_ at the three interfaces. The two pressure‐driven processes are described by

(5) $$\begin{array}{c} \frac{dx_{i}}{dt}={\left(\frac{dx_{i}}{dt}\right)}_{\exp}+{\left(\frac{dx_{i}}{dt}\right)}_{\mathrm{equ}}\\ \;=\mu_{i}\left[\Theta \left(p_{\mathrm{tag}}-p_{i}\right)\left(p_{\mathrm{tag}}-p_{i}\right)+\left(p_{\mathrm{eff}}-p_{i}\right)\right]\;\left(i=\mathrm{cyt},\mathrm{lum}\right). \end{array}$$

The Heaviside step function θ(*x*) in Eqn (5) indicates that TAG filling of the pLD is irreversible, i.e. the pLD cannot reduce its volume by pumping TAG back into the cap monolayers. The $\mu_{i}$ values are empirical rate parameters having the dimension μm·s^−1^·kPa^−1^. The solvation pressure *p*_tag_ is given by Eqn (3). The effective pressure *p*_eff_ is chosen such that pressure equilibration proceeds at constant volume Ω of the pLD:

(6) $$\frac{\mathrm{d\Omega}}{dt}=\sum_{i}\frac{\mathrm{d\Omega}}{dx_{i}}{\left(\frac{dx_{i}}{dt}\right)}_{\mathrm{equ}}=0.$$

Relation (6) implies

(7) $$p_{\mathrm{eff}}=\frac{\mu_{\mathrm{cyt}}\frac{\mathrm{d\Omega}}{dx_{\mathrm{cyt}}}p_{\mathrm{cyt}}+\mu_{\mathrm{lum}}\frac{\mathrm{d\Omega}}{dx_{\mathrm{lum}}}p_{\mathrm{lum}}+\mu_{\mathrm{int}}\;p_{\mathrm{int}}}{\mu_{\mathrm{cyt}}\frac{\mathrm{d\Omega}}{dx_{\mathrm{cyt}}}+\mu_{\mathrm{lum}}\frac{\mathrm{d\Omega}}{dx_{\mathrm{lum}}}+\mu_{\mathrm{int}}}.$$

For the expansion of the pLD along inter‐monolayer space, we use Darcy's law, which states that the volume flow of a fluid (here: oil composed of TAG and other hydrophobic molecules as cholesterol esters) in a porous medium (here: inter‐monolayer space constituted by fatty‐acid tails of PLs and integral membrane proteins) is proportional to the applied pressure gradient:

(8) $$\begin{array}{c} \frac{d\Omega_{\mathrm{int}}}{dt}=\frac{\partial \Omega }{\partial x_{\mathrm{int}}}\frac{dx_{\mathrm{int}}}{dt}\\ \;=\nu_{\mathrm{int}}\left[\Theta \left(p_{\mathrm{tag}}-p_{\mathrm{int}}\right)\left(p_{\mathrm{tag}}-p_{\mathrm{int}}\right)+\left(p_{\mathrm{eff}}-p_{\mathrm{int}}\right)\right]. \end{array}$$

The volume‐flow rate ν_int_ has the dimension nm^3^·kPa^−1^·s^−1^ and depends on the permeability constant *k*_int_ of the inter‐monolayer space and the viscosity $\varsigma_{\mathrm{TAG}}$ of the TAG fluid, $\nu_{\mathrm{int}}=k_{\mathrm{int}}/\varsigma_{\mathrm{TAG}}$ [10]. *p*_int_ denotes the lateral counter‐pressure of the bilayer. Rearranging Eqn (8) and employing $\partial \Omega_{\mathrm{int}}/\partial x_{\mathrm{int}}=2\mathrm{\pi d}x_{\mathrm{int}}$ gives

(9) $$\begin{array}{c} \frac{dx_{\mathrm{int}}}{dt}={\left(\frac{dx_{\mathrm{int}}}{dt}\right)}_{\exp}+{\left(\frac{dx_{\mathrm{int}}}{dt}\right)}_{\mathrm{equ}}\\ \;=\frac{1}{x_{\mathrm{int}}}\frac{\nu_{\mathrm{int}}}{2\pi d}\left[\Theta \left(p_{\mathrm{tag}}-p_{\mathrm{int}}\right)\left(p_{\mathrm{tag}}-p_{\mathrm{int}}\right)+\left(p_{\mathrm{eff}}-p_{\mathrm{int}}\right)\right]. \end{array}$$

From Eqn (9) follows that the parameter μ_int_ in Eqn (5) decreases with increasing lateral extension according to $\mu_{i}=\frac{1}{x_{\mathrm{int}}}\frac{\nu_{\mathrm{int}}}{2\pi d}$.

In a tense‐free bilayer, the average lateral pressure is zero. However, if the reservoir monolayers have a finite surface area as is the case for GUVs, the net flow of PLs from the reservoir monolayers into the caps of the pLD results in a PL depletion in the reservoir monolayers, which is accompanied by an increase in the line tension (see Eqn 19). According to a theoretical model [26], *p*_int_ linearly rises with the surface tension of the bilayer. Moreover, lateral expansion of the oil drop increases the hydrostatic pressure of the mobile water in the bulk phase [27]. Therefore, for simulations of pLD formation in the bilayer of GUVs, the lateral pressure *p*_int_ of the bilayer was chosen as a linear function of lateral extension,

(10) $$p_{\mathrm{int}}=\lambda_{\mathrm{int}}x_{\mathrm{int}}.$$

$\lambda_{\mathrm{int}}$ is a phenomenological parameter defining the increase of the bilayer pressure per lateral expansion length of the pLD.

### Expansion of the pLD towards the cytosolic and luminal space

The expansion of the initially flat monolayer surface *A*_o_ to the spherical surfaces *A*_*i*_ (*i* = cyt, lum) of the two caps is accomplished in two different ways: (a) Pressure‐driven elasto‐mechanical bending and stretching and (b) uptake or removal of membrane particles, such as PLs and membrane proteins. The inward‐directed normal pressure of a spherical bulge with radius *r* and surface area *A* is given by the Laplace equation

(11) $$p=\frac{2T}{r}=\frac{2}{r}\left(T_{S}+T_{A}+T_{B}\right).$$

The surface tension *T* is made up of three contributions: the interfacial phospholipid‐solvent tension *T*_S_, the tension *T*_A_ generated by extension of the surface area *A*, and the tension *T*_B_ generated by bending of *A*. *T*_S_ of the ER membrane is negligibly small (≈ 0.01 mm·m^−1^ [28]) compared to a typical LD surface tension of about 1 mm·m^−1^ [5] and thus will be disregarded in the following.

The extension d*A* = *A* − *A*_o_ of the surface A can be sufficiently well described [29] by the linear relation

(12) $$T_{A}=\kappa_{a}\left|\frac{A-A_{o}}{A_{o}}\right|=\left|\frac{\mathrm{dA}}{A_{o}}\right|.$$

κ_a_ is the area extension modulus, which for PL monolayers amounts to about 250 mN·m^−1^ [14]. Notably, the relative change d*A* may not exceed 5–8% without disruption of the monolayer [30]. The physical and chemical properties of the monolayer may change during expansion due to the incorporation of additional lipids. To incorporate such effects in the calculation of the surface tension, we compute the elementary force *f*_PL_ required for changing the distance *d*_PL_ between neighboured PLs. Assuming a harmonic potential, we may put

(13) $$f_{\mathrm{pl}}=k\left|d_{\mathrm{pl}}^{2}-d_{\mathrm{plo}}^{2}\right|,$$

where *d*_plo_ is the next‐neighbour distance of PLs in the relaxed, non‐expanded monolayer. If the PLs are evenly distributed across the surface area *A*, their centres are separated by the distance

(14) $$d_{\mathrm{pl}}=\sqrt{\frac{2}{\pi }}\sqrt{\frac{1}{\rho_{\mathrm{pl}}}},$$

where *n*_PL_ is the number of PLs in *A* and ρ_pl_ = *n*_pl_/*A* is the surface density. Relation (14) is easily obtained by representing the share of a single PL in the surface area *A* by a circle, the radius of which is chosen such that the sum of circle areas equals the surface area *A*. Substituting (14) into (13) results in

(15) $$f_{\mathrm{pl}}=\kappa_{a}\left|\frac{1}{\rho_{\mathrm{pl}}}-\frac{1}{\rho_{o}}\right|,$$

with ρ_o_ being the normal PL density. The surface area α of a single PL amounts to approximately 0.5 nm^2^ [16], although it may differ in the head and tail region depending on the chemical structure of the PL [17]. In this study, we will put ρ_o_ = 1/α = 2 × 10^6^ μm^−2^. The total surface tension *T*_A_ is obtained by adding the elementary force *f*_pl_ across the number *n*_pl_ of PLs and dividing this sum through the surface area *A*:

(16) $$T_{A}=\frac{n_{\mathrm{PL}}}{A}f_{o}=\kappa_{a}\rho_{\mathrm{pl}}\left|\frac{1}{\rho_{\mathrm{pl}}}-\frac{1}{\rho_{o}}\right|=\kappa_{a}\left|1-\frac{\rho_{\mathrm{pl}}}{\rho_{o}}\right|.$$

Relation (16) states that no elasto‐mechanical force is required for the expansion of the monolayer surface *A* as long as the uptake of additional PLs is large enough to keep the PL density $\rho_{\mathrm{pl}}$ at the reference value $\rho_{o}$ of the unstressed monolayer. Relation (16) is a generalisation of Relation (12): If the monolayer surface *A* does not uptake, release, or exchange PLs during expansion, the PL density is $\rho =\rho_{o}A_{o}/A$, and Relation (16) becomes identical with Relation (12) for small deviations d*A* = (*A* − *A*_o_)/*A*_o_ < 1.

To include the contribution of both bending and stretching to the surface tension, we exploit the fact that bending a surface of finite thickness and positive curvature means stretching the outer surface and compressing the inner surface. We thus subdivide the tension generated by the force between adjacent PLs into three contributions resulting from the forces acting within the head‐group region (h), mid‐layer region (m), and fatty‐acid tail region (t) of the monolayer:

(17) $$T_{i}=\kappa_{i}\left|1-\frac{\rho_{\mathrm{pl}}^{i}}{\rho_{o}}\right|\;\left(i=h,m,t\right).$$

κ_*i*_ (*i* = h, m, t) are the respective elastic moduli whose values differ as different forces dominate in the three monolayer regions: electrostatic dipole–dipole forces of the polar head‐groups among each other and with water dipoles, and hydrophobic forces between the PL chains in the bulk phase constituted by the FA chains, which interact at their methyl ends with the lipids of the pLD. The surface area *A* and the curvature 1/*r* are measured in the middle of the monolayer, *A* = *A*_m_, *r* = *r*_m_. For spherical surface areas, it holds

(18) $$\begin{array}{c} \frac{A_{h}}{A_{m}}=\frac{{\left(r+d_{m}/2\right)}^{2}}{r^{2}}\approx 1+\frac{d_{m}}{r}\\ \frac{A_{t}}{A_{m}}=\frac{{\left(r-d_{m}/2\right)}^{2}}{r^{2}}\approx 1-\frac{d_{m}}{r} \end{array}$$

as long as *d*_m_/*r* ≪ 1. Using the Relation (18), the PL densities $\rho_{\mathrm{pl}}^{h}$ and $\rho_{\mathrm{pl}}^{t}$ in (17) can be expressed through the surface density $\rho_{\mathrm{pl}}=n_{\mathrm{pl}}/A$:

(19) $$\begin{array}{c} T=T_{h}+T_{m}+T_{t}\\ T_{h}=\kappa_{h}\left|1-\frac{\rho_{\mathrm{pl}}}{\rho_{o}}\left(1-\frac{d_{m}}{r}\right)\right|\\ T_{h}=\kappa_{m}\left|1-\frac{\rho_{\mathrm{pl}}}{\rho_{o}}\right|\\ T_{t}=\kappa_{h}\left|1-\frac{\rho_{\mathrm{pl}}}{\rho_{o}}\left(1+\frac{d_{m}}{r}\right)\right|. \end{array}$$

The contributions of bending and stretching (or compression, respectively) to the surface tension are coupled via the PL density ρ_pl_, although this coupling is weak because ρ_pl_/ρ_o_ ≈ 1. If the tension is generated only by area extension, i.e. 1/*r* = 0, Eqns (19, 12) have to yield identical tensions. This implies the relation $\kappa_{h}+\kappa_{m}+\kappa_{t}=\kappa_{a}$ for the sum of the elastic moduli. An estimate of the elastic moduli for bending is obtained by putting $\rho_{\mathrm{pl}}=\rho_{o}$ in Eqn (19):

(20) $$T_{B}=T\left(\rho =\rho_{o}\right)=\left(\kappa_{h}+\kappa_{t}\right)\frac{d}{\left|r\right|}.$$

The bending tension *T*_B_ is equivalent with a bending force *F*_B_ = *T*_B_ *d*_m_ with *d*_m_ being the thickness of the monolayer. On the other hand, the bending force *F*_B_ obeys the well‐known relation *F*_B_ = 2π*B*/*r* [31] with *B* being the bending rigidity of the monolayer. Thus, $\kappa_{h}+\kappa_{t}=2\pi B/d_{m}^{2}$. With a typical binding rigidity of $B=4\times 10^{-20}\mathrm{Nm}$ [32] and *d*_m_ = 2 nm, we get a rough estimate $\kappa_{h}+\kappa_{t}$ ≈ 60 mN·m^−1^. We thus will put $\kappa_{h}=\kappa_{t}$ = 30 mN·m^−1^ for both monolayers in all simulations.

For the flat (1/*r* = 0) reservoir monolayer A*, the general Eqn (19) reduces to

(21) $$T^{*}\equiv T_{A}^{*}=\kappa_{a}\left|1-\frac{\rho^{*}}{\rho_{o}}\right|.$$

According to Relation (21), any change in the PL density of the reservoir monolayers is accompanied by a change in the surface tension.

### Kinetics of membrane lipids

Temporal changes in the PL surface densities in the cap monolayers are governed by Eqn (22):

(22) $$\frac{d\rho_{\mathrm{pl}}^{i}}{dt}=-\frac{n_{\mathrm{pl}}^{i}}{dt}\sum_{i}\frac{\partial A}{\partial x_{i}}\frac{dx_{i}}{dt}+\frac{1}{A_{i}}\frac{dn_{\mathrm{pl}}^{i}}{dt}\;\left(i=\mathrm{cyt},\mathrm{lum}\right).$$

The first term on the right‐hand side describes density changes caused by elasto‐mechanical changes in the surface area. The time derivatives of the principal coordinates *x*_*i*_ are given by Eqn (5). The second term describes density changes caused by changes in the number of PLs in the monolayer. These may result from the equilibration of PL densities between the cap monolayer and the adjacent reservoir monolayer and – in the cytosolic monolayer – *de novo* PL synthesis catalysed by enzymes located on the monolayer surface. For the inclusion of these two kinetic processes, we use simple mass‐action rate equations:

(23) dδnplcytdt=kdcytAcytppl*cyt−ρplcyt+ksplcytAcytΘρ0−ρplcytρ0−ρplcytdδnpl*cytdt=−kdcytAcytppl*cyt−ρplcyt+kspl*cytAcyt*Θρ0−ρpl*cytρ0−ρpl*cytdδnpllumdt=kdlumAlumppl*lum−ρpllumdδnpl*lumdt=−kdlumAlumppl*lum−ρpllum.

$\delta n_{\mathrm{pl}}^{i}$ (*i* = cyt, lum) refers to the number of PLs that are *additionally* taken up or released by the cap‐covering monolayer (*i*), $k_{d}^{i}$ denotes the rate constant for the equilibration of the PL densities in *A*_*i*_ and $A_{i}^{*}$. ${\mathrm{ks}}_{\mathrm{pl}}^{i}$ and ${\mathrm{ks}}_{\mathrm{pl}}^{*i}$ are the rate constants for the synthesis of PLs in the cap and reservoir monolayers per unit area 1 μm^2^, respectively. The rate equation for PL synthesis is based on the assumption that the uptake of additional PLs into the monolayer will cease when the normal PL density ρ_o_ is reached. Regarding the absolute magnitude of the rate constants in Eqn (23), 31P spin–lattice (T1) and spin–spin (T2) PL relaxation measurements in synthetic bilayers [33] yielded relaxation time of 10–100 ms^−1^ corresponding to values of 1–10 s^−1^ for $k_{d}^{i}$. The rate constants for PL synthesis per unit area (= 1 μm^2^) monolayer should have the same order of magnitude. This can be concluded from experiments in yeast [34] demonstrating that the formation of autophagosomes with a diameter of 1 μm was reduced by more than 50% within 5 min at knock‐out of the acyl‐CoA synthase (Faa1). This implies that local channelling of PLs into the membrane of ER‐derived growing organelles may generate more than 1 μm^2^ surface area (≈ 2 × 10^6^ PLs) per minute.

### Kinetics of TAG

Analogous to Eqn (22), the temporal change in the TAG surface density $\rho_{\mathrm{tag}}^{m}$ in the cytosolic cap monolayer is given by

(24) $$\frac{d\rho_{\mathrm{tag}}}{dt}=-\frac{\rho_{\mathrm{tag}}}{A_{\mathrm{cyt}}}\sum_{i}\frac{\partial A_{\mathrm{cyt}}}{\partial x_{i}}\frac{dx_{i}}{dt}+\frac{1}{A_{\mathrm{cyt}}}\frac{d\rho_{\mathrm{tag}}}{dt}.$$

Temporal changes in $n_{\mathrm{tag}}$, the number of TAG molecules in *A*_cyt_, are caused by differences between the rate of TAG uptake vu_tag_ and the transition rate vt_tag_ into the pLD. Due to the presumed incompressibility of the pLD, the transition rate vt_tag_ is proportional to the rate of volume change:

(25) $$\frac{d\rho_{\mathrm{tag}}}{dt}={\mathrm{vu}}_{\mathrm{tag}}-{\mathrm{vt}}_{\mathrm{tag}}={\mathrm{ku}}_{\mathrm{tag}}\left(1-\frac{\rho_{\mathrm{tag}}}{\rho_{\mathrm{tag}}^{\#}}\right)-\frac{1}{\omega }\frac{\mathrm{d\Omega}}{dt}.$$

The rate constant ku_tag_ represents the maximal rate of TAG uptake per $\mu m^{2}$ area of the monolayer whereby no distinction is made between TAG that is synthesised in the cap monolayer and TAG, which flows into the cap monolayer from the reserve monolayer. $\rho_{\mathrm{tag}}^{\#}$ denotes the maximal TAG density in the monolayer.

### LD formation

The cytosolic or luminal caps of the pLD transform into a new LD when one of them undergoes energetic instability [21, 22]. The most critical part of the expanding monolayer, experiencing the highest surface tension, is the sharply curved edge at the bilayer/cap transition zone (see Fig. 1B). The radius of curvature *r*_e_ of the narrowest cross‐section is put to $r_{e}=L_{c}/\left(\pi -\psi \right)$ where the elastic length *L*_c_ is the lateral distance along which the monolayer is able to relax the elastic stress caused by bending. The value of elastic length can be estimated from the relation $L_{o}=\sqrt{B/T}$ [23, 24]. With *B* = 10^−20^ Nm and *T* = 1 mN·m^−1^, we get *L*_o_ ≈ 10 nm. The maturation time $T_{m}$ of the LD is defined by the time point at which the edge tension reaches the rupture tension:

(26) $$\begin{array}{c} \eta \left(T_{m}\right)=0\\ \eta \left(t\right)=1-\frac{T_{e}}{T_{r}}. \end{array}$$

The edge tension *T*_e_ is given by Eqn (17) with *r* = *r*_e_. *T*_r_ is the rupture tension at which the connection between the cap monolayer and the reservoir monolayer is broken. Typical values of *T*_r_ are in the range of 5–12 mN·m^−1^ as determined with single‐bilayer vesicles made from poly‐unsaturated PC bilayers [30, 35]. Depending on whether the condition η=0 is met first for the cytosolic or the luminal cap, either a cytosolic or a luminal LD is detached from the bilayer.

The rate of TAG export from the ER membrane into the cytosol per single LD is given by the export rate

(27) $$v_{\text{export}}=\frac{{\mathrm{TAG}}_{\mathrm{cyt}}\left(T_{m}\right)}{T_{m}}$$

### Synthetic droplets embedded in the bilayer of a giant unilamellar vesicle (GUV)

**Fig. 2:**
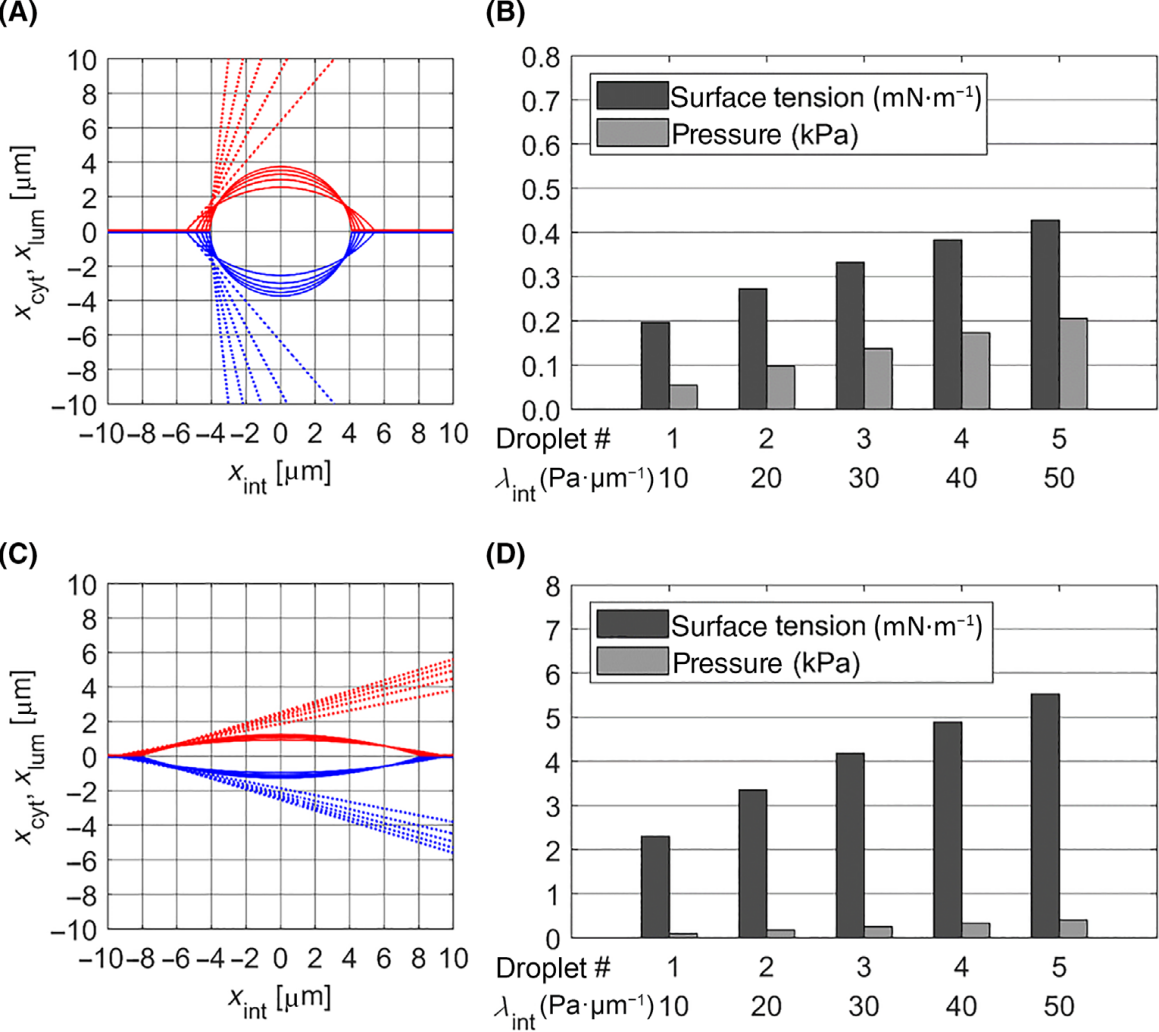
Lipid lenses in closed bilayers mimicking synthetic pLDs in GUVs. A fixed amount of 2.7 × 10^11^ TAG molecules corresponding to the TAG store of a spherical LD with a diameter of about 8 μm was channelled into a bilayer with surface area *A*_tot_ = 2.5 × 10^4^ μm^2^ corresponding to the surface area of a GUV with a diameter of 36 μm. Red and blue lines indicate the cytosolic and luminal monolayers. The parameter λ_int_ determining the increase of the lateral bilayer pressure *p*_int_ with increasing lateral extension *x*_int_ of the pLD (see Eqn 9) was varied. See Table 1 for the values of the other model parameters. (A) Shape of pLDs at λ_int_ = 10, 20, 30, 40, 50 Pa·μm^−1^. The contact angles ψ_cyt_ and ψ_lum_ of the pLDs are enclosed by the red and blue dotted lines and the horizontal bilayer surface. pLD #3 generated with λ_int_ = 30 Pa·μm^−1^ was chosen for further simulations shown in Fig. 3. (B) Surface tension and effective pressure of the pLDs shown in (A). (C) Shapes of pLDs at blocked PL exchange between cap and adjacent reservoir monolayers (*k*_d_ = 0 in Eqn 23). (D) Surface tension and effective pressure of the pLDs shown in (C).

An attractive method to study the influence of membrane parameters on the size and shape of the membrane‐harboured lipid lenses is offered by artificial pLDs either embedded in the bilayer of GUVs or generated as so‐called droplet interface bilayers based on micrometric adhesive emulsion droplets [12]. To recapitulate the experimental results obtained with such *in vitro* systems, we first simulated the incorporation of a fixed amount of TAG into the inter‐monolayer space of a fully symmetric bilayer (see Fig. 2).

With all mechanical and kinetic parameters fixed (cf. Table 1), the final shape of the pLD depends only on the model parameter $\lambda_{\mathrm{int}}$ determining the increase of the lateral bilayer pressure *p*_int_ with increasing lateral extension of the pLD (cf. Eqn 10).

**Table 1:** Parameters of the standard model. Parameter values hold for both the cytosolic and luminal monolayer (symmetric bilayer). Deviating values in some of the simulations are explicitly stated in the main text and the figure legend.

Parameter	Meaning	Value
μ_cyt_ (μm·s^−1^·kPa^−1^)	Expansion rate cytosol	1
μ_lum_ (μm·s^−1^·kPa^−1^)	Expansion rate lumen	1
ν_int_ (nm^3^·s^−1^·kPa^−1^)	Rate of lateral volume flow	1
κ_m_ (mN·m^−1^)	Elastic modulus mid‐plane	190
κ_h/t_ (mN·m^−1^)	Elastic modulus head/tail	30
A* (μm^2^)	Surface area reservoir monolayer	2.5 × 10^3^
*K*_equ_	TAG equilibrium membrane:pLD	20
ρ_ο_ (μm^3^)	PL density in non‐expanded monolayer	1.996 × 10^6^
ku_tag_ (min^−1^·μm^−2^)	Maximal uptake rate of TAG	1.2 × 10^8^
*k*_d_ (s^−1^)	Lateral PL flow rate	10
λ_int_ (Pa·μm^−1^)	Lateral pressure increase	0.03
*T*_r_ (mN·m^−1^)	Rupture tension	10
α (nm^2^)	Average phospholipid surface area	0.5
ω (nm^3^)	Molecular volume TAG	1.0

Restricting the value of this parameter to a range between 10 and 50 Pa·μm^−1^, the simulations yielded stationary pLDs with horizontal diameters of 4–6 μm and surface tensions between 0.2 and 0.45 mN·m^−1^. These values are in agreement with values observed in symmetric PC bilayers of GUVs filled with triolein [4]. Increasing values of $\lambda_{\mathrm{int}}$ resulted in more spherical pLDs with higher pressure and surface tension. The generation of a lateral pressure *p*_int_ of the bilayer was essential in these simulations. Otherwise, a steady state would only be achieved by a homogeneous distribution of TAG throughout the interlayer space of the bilayer. Shapes of pLDs as those shown in Fig. 2A with contact angles ψ > 90° have been typically observed for pLDs embedded in GUVs with various PL compositions. In contrast, bud‐like shapes with contact angles ψ < 90° have not been obtained without additionally enforcing an increase of the surface tension of one of the two monolayers, e.g. by aspiration with micropipettes [3, 4] or changes of the osmotic pressure [21, 36].

To demonstrate the importance of PL transfer between the reservoir and cap monolayers in keeping the surface tension low and thus allowing a substantial expansion of the surface area, the same simulations were performed with blocked lateral PL flow, that is, setting *k*_d_ = 0 in Eqn (23). A flat, laterally extended lens was developed (Fig. 2C). The surface area of the caps expanded only marginally by about 2% compared to the reference surface area. The surface tensions went up to values of 2–5 mN·m^−1^, which are about tenfold higher than in the case of unrestricted lateral PL flow (Fig. 2B,C).

**Fig. 3:**
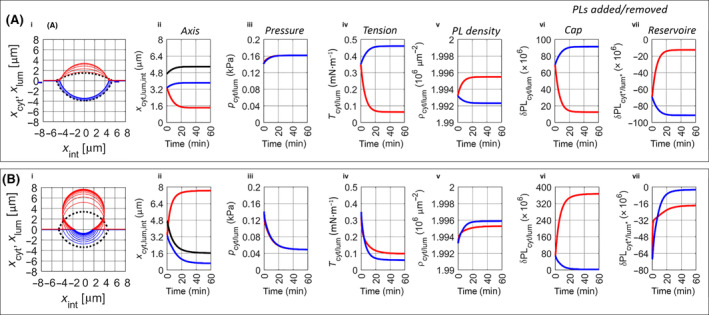
Shape changes of the pLD induced by asymmetric surface tensions. (A) (i) Transition of the pLD #3 from the initial steady‐state conformation (indicated by black dotted lines) into a new steady‐state induced by the application of an additional pressure to the cytosolic surface of the bilayer. Red and blue lines indicate the cytosolic and the luminal monolayer. Thin lines mark the conformation of the pLD at intervals of 1 min. An additional pressure Δ*p* of 0.15 kPa was exponentially switched on with a half‐time of 60 s, $\Delta p=0.15\times \left(1-\exp\left(-t/60\right)\right)$. The red and blue lines in panels (ii)–(vii) show the temporal changes of selected model parameters. (ii) Principal axis of the pLD; *x*_int_ is represented by the black line. (iii) Effective pressure of the pLD. (iv) Surface tension of the cap monolayers. (v) PL density of the cap monolayers. (vi) Additional PLs added to or removed from the cap monolayers. (vii) Additional PLs added to or removed from the adjacent reservoir monolayers. (B) (i) Transition of the pLD induced by supply of 1.8 × 10^8^ PLs to the external medium mimicking the cytosol. For a total surface area of 2.5 × 10^4^ μm^2^ of the bilayer, this corresponds to a surface density of ρ_s_ = 7.2 × 10^3^ μm^−2^. The PL incorporation rate was put to vs_pl_ = ρ_s_ × ks_pl_ with ks_pl_ = 10^5^ s^−1^·μm^−2^. The model variables depicted in panels (ii)–(vii) are identical to those in (A).

The pLD#3 (cf. Fig. 2A) was taken as the starting point for further simulations where changes in the size and shape of the pLD were induced by mechanical stress or supply of additional PLs. The simulations shown in Fig. 3A mimic experiments where a negative pressure of 0.15 kPa was applied to the cytosolic monolayers *A*_cyt_ and $A_{\mathrm{cyt}}^{*}$. This resulted in a shift of the pLD in the direction of the lumen. In the new stationary state, the pressure of the pLD was marginally increased by about 10% but the tension of the luminal monolayer was about 8‐fold higher than the tension of the cytosolic monolayer. Flattening of the cytosolic cap was paralleled with an outflow of about 4 × 10^7^ PLs to the reservoir monolayer, which almost completely restored the PL density to the normal density ρ_o_. In contrast, the PL density of the luminal monolayer continued to decrease despite an influx of about 2 × 10^7^ PLs from the reservoir monolayer. The shift of the pLD towards the luminal space was not accompanied by the adoption of a budding conformation; the luminal contact angle $\psi_{\mathrm{lum}}$ remained essentially the same.

Figure 3B shows a simulation demonstrating that the supply of additional PLs to the cytosolic space of the GUV‐embedded pLD promotes an outward pLD drift. In the absence of TAG supply, a fixed amount of 3.5 × 10^8^ PLs was added to the external space at time *t* = 0, corresponding to a density of 1.4 × 10^4^ μm^−2^ surface area of the GUV. The rate constants for the uptake of PLs into the cytosolic monolayers were set to ${\mathrm{ks}}_{\mathrm{pl}}^{\mathrm{cyt}}={\mathrm{ks}}_{\mathrm{pl}}^{*\mathrm{cyt}}$ = 2 × 10^6^ s^−1^ to allow the new steady state to be reached within 60 min. Due to the large PL‐water partition coefficient (log(*K*) = 3–4, [37]), the incorporation of PLs into the cytosolic monolayers is quasi irreversible and thus can be described by the kinetic Eqn (23). The uptake of additional PLs increased the PL density of the cytosolic cap and reservoir monolayer and thus lowered surface tension and pressure. About 80% of added PLs were incorporated into the cytosolic cap monolayer. Pressure equilibration caused a shift of the pLD towards the cytosol resulting in an additional expansion of the cytosolic cap and shrinking of both the luminal cap and lateral disk. Almost all PLs initially taken up by the luminal monolayer from the reservoir monolayer during TAG loading of the pLD (≈ 8 × 10^7^) returned to the reservoir monolayer. In contrast to shape changes induced by mechanical pressure, the cytosolic cap adopted a bud‐like conformation with ψ_cyt_ < 90°.

Taken together, the above simulations recapitulate findings obtained with artificial LDs [5, 6, 7, 12, 38]: An asymmetry in the energy of the cap monolayers may give rise to a shift of the pLD into the direction of the monolayer with lower surface energy. In particular, the shift induced by the uptake of additional PLs into one of the two pLD‐covering monolayers promotes the adoption of a more spherical, bud‐like shape of the respective cap. Owing to the very low elasto‐mechanical expandability of PL monolayers (3–5%), significant changes in the surface areas of the cytosolic and luminal cap require the exchange of PLs with the adjacent reservoir monolayers.

### Formation of LDs *in vivo*

The synthesis of LDs at the ER membrane differs in many aspects from the embedding of artificial LDs in a synthetic bilayer: (a) The ER membrane contains a multitude of different PLs and peripheral and integral membrane proteins influencing the rate of the lateral PL flow and the elastic properties of the monolayers. (b) The cytosolic monolayer of growing pLDs has permanent access to newly synthesised TAG and PLs. (c) Growth of the pLD does not reach a steady state. Rather, dynamical expansion of the cytosolic cap of the pLD leads to a more and more bud‐like structure until the edge tension exceeds a critical value above which the connection between the cap monolayer and the reservoir monolayer is broken. (d) Compared to large synthetic LDs with diameters of 5–20 μm, nascent LDs freshly synthesised at the ER of various cell types are much smaller with typical diameters of 30–500 nm [9, 15]. The period for the synthesis of a new LD was reported to be in the range of 5–15 min [9, 39].

The *in vivo* simulations were carried out under four assumptions: (a) Formation of a new pLD starts at a distinct site of the ER bilayer with coordinate *x*_int_ = 0. (b) The surface areas $A_{\mathrm{cyt}}^{*}$ and $A_{\mathrm{lum}}^{*}$ of the reservoir monolayers are large compared to the surface areas of the cap monolayers (2500 μm^2^). (c) The uptake rate constant ku_tag_ of TAG per unit surface of the cytosolic cap monolayer was kept constant. (d) Trans‐bilayer movement of PLs (flip‐flop) was neglected.

**Fig. 4:**
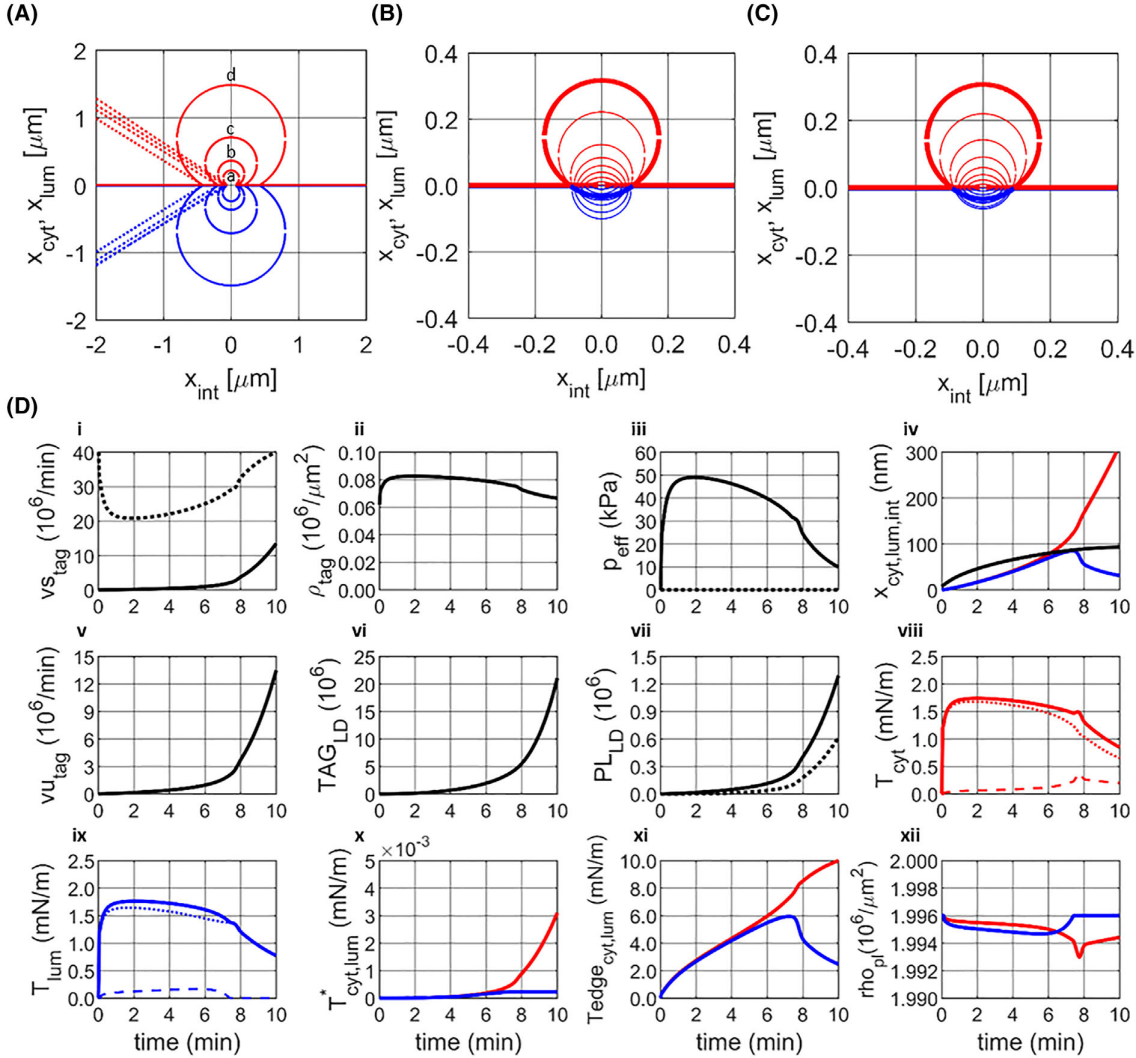
Simulated LD formation. (A) LD formation in a fully symmetric bilayer: Shape and size of the pLD at the time point of LD detachment (= maturation time, *T*_m_) for different values of the lateral expansion rate ν_int_: (a) ν_int_ = 0.1 nm^3^·s^−1^·kPa^−1^, diameter = 225 nm, *T*_m_ = 24 min. (b) ν_int_ = 1 nm^3^·s^−1^·kPa^−1^, diameter = 390 nm, *T*_m_ = 15 min. (c) ν_int_ = 10 nm^3^·s^−1^·kPa^−1^, diameter = 765 nm, *T*_m_ = 21 min. (d) ν_int_ = 100 nm^3^·s^−1^·kPa^−1^, diameter = 1637 nm, *T*_m_ = 40 min. Red and blue lines indicate the cytosolic and the luminal monolayer. The contact angle enclosed by the dotted lines and the bilayer mid‐plane at the time of LD detachment was 32° for all pLDs. (B) Time‐dependent LD formation in an elastically asymmetric bilayer: The elastic modulus κ_m_ was decreased by 10% (= −20 mN·m^−1^) for the cytosolic monolayer and increased by 10% (= +20 mN·m^−1^) for the luminal monolayer, i.e. Δκ_m_ = −40 mN·m^−1^. The lateral volume flow rate was set to ν_int_ = 1 nm^3^·s^−1^·kPa^−1^ as for the second smallest pLD in (A). Thin lines indicate shape changes at intervals of 1 min. Bold lines indicate the shape of the pLD at *T*_m_ = 10 min where detachment of the cytosolic cap under the formation of an LD occurs. For the temporal change of the principal coordinates, see panel (C). Note that the cytosolic and the luminal cap expand at the same rate up to *t* = 7.5 min, after which the luminal cap contracts while the cytosolic cap continues to expand. (C) LD formation in a kinetically asymmetric bilayer where the rate constant kd_lum_ for the lateral flow of PLs in the luminal monolayer was decreased to 50% of the reference value, kd_lum_ = 0.5 × kd_cyt_ = 5 s^−1^. Bold lines indicate the shape of the pLD at *T*_m_ = 9.8 min where detachment of the cytosolic cap as LD occurs. (D) Time‐course of selected model parameters for the formation of the LD shown in panel (C). Red and blue lines refer to the cytosolic and luminal parameters: (i) Bold line: Total rate of TAG. Dotted line: Rate of TAG supply per unit area (= 1 μm^2^) of the cytosolic cap monolayer. (ii) TAG density of the cytosolic cap monolayer. (iii) Effective pressure of the pLD (bold line) and lateral pressure *p*_int_ (dotted line). (iv) Time‐course of the three principal coordinates *x*_cyt_ (red), *x*_lum_ (blue), and *x*_int_ (black). (v) Uptake rate of TAG into the pLD. (vi) TAG content of the cytosolic cap monolayer. (vii) Bold line: Total PL content of the cytosolic cap monolayer. Dotted line: Share of PLs taken up from the cytosolic reservoir monolayer. (viii) Tension of the cytosolic cap monolayer (bold line), bending tension (dotted), and stretch tension (dashed). (ix) Tension of the luminal cap monolayer (bold line), bending tension (dotted), stretch tension (dashed). (x) Tension of the cytosolic reservoir monolayer and luminal reservoir monolayer. (xi) Edge tension of the cytosolic monolayer and luminal monolayer. (xii) PL density of the cytosolic cap monolayer and luminal cap monolayer.

First, the expansion of a fully symmetric bilayer with identical kinetic and elastic parameters of both monolayers was simulated. To generate LDs with a diameter of 300–400 nm within 5–15 min, the rate constant $\nu_{\mathrm{int}}$ for the lateral volume flow of TAG within the bulk bilayer had to be set to values in the range of 0.1–100 nm^3^·s^−1^·kPa^−1^. As would be dictated by the perfect symmetry of the two monolayers, the simulations resulted in symmetrical pLDs (see Fig. 4A). The monolayers of both caps reached the rupture tension within the same maturation time of 24–40 min. The magnitude of the lateral volume flow rate $\nu_{\mathrm{int}}$ strongly controls the final size of the symmetric pLD. Increasing the value of this parameter from 0.1 to 100 nm^3^·s^−1^·kPa^−1^, the terminal diameter of the caps increased about 7‐fold from 225 to 1637 nm. With *T*_m_ = 15 min, the maturation time was lowest at $\nu_{\mathrm{int}}$ = 1 nm^3^·s^−1^·kPa^−1^. However, in total, the variation in the maturation times for the cases shown in Fig. 4A was only a factor of 2.

As shown in the above simulations of GUV‐embedded pLDs and demonstrated in several experiments, the directional expansion of the pLD into the cytosol requires keeping the tension of the cytoplasmic monolayer lower than that of the luminal monolayer. This can be achieved by altering the elasto‐mechanical or kinetic properties of the cap monolayers, for example by changing the PL composition [40] or by adsorption of membrane proteins. We simulated these possible effects by changing the elastic moduli of the monolayers and the PL exchange rates between the luminal cap and reservoir monolayer. Both mechanisms ensure a preferential expansion of the cap whose monolayer has a relatively lower surface tension. Figure 4B illustrates the time‐resolved expansion of a pLD in an elastically asymmetric bilayer. The elastic modulus κ_m_ of the cytosolic monolayer was 10% lower and that of the luminal monolayer was 10% higher than the reference value of 190 mN·m^−1^. This asymmetry in the elastic properties of the monolayers caused an almost exclusive expansion of the cytosolic monolayer. The relative proportion of the volume of the luminal cap was only 2% at the time of LD detachment. The smaller the difference in the elastic moduli was chosen, the slower the pLD expanded but the terminal budding configuration adopted by the pLD before detachment remained the same. Hence, an arbitrarily small (in a mathematical sense) difference in the elastic constants of the complementary monolayers is sufficient to enforce exclusive expansion of the pLD in the direction of either cytosol or lumen.

In a further simulation, the values of the elastic moduli of the two monolayers were identical, but the flow rate of the PLs in the luminal monolayer was reduced to 50% of the reference value (see Fig. 4C). This kinetic asymmetry produced the same growth characteristic as obtained for the elastic asymmetry: The luminal cap initially grows as fast as the cytosolic cap, but then begins to shrink when the curvature of the cytosolic monolayer falls below a critical value. At the time of LD detachment, the residual volume of the luminal cap amounts to only 2–3% of the LD volume. Note that this proportion depends on the value of the rupture tension (here: 10 mN·m^−1^): The larger the value of the rupture tension, the smaller the size of the residual TAG fraction remaining in the bilayer after LD detachment.

Figure 4D shows more details of the formation of the LD in a kinetically asymmetric bilayer. As can be taken from the timely equidistant contour lines in Fig. 4A and the temporal changes of the principal coordinates in Fig. 4Div, one may distinguish between two phases of pLD growth. In the first (slow growth) phase of about 7 min, lateral spreading of TAG along the inter‐monolayer space of the bilayer dominates the expansion. Both caps expand at an equal rate, their surface tensions are dominated by the contribution of bending. During this first phase, the curvature 1/*R*_cyt_ of the monolayers steadily decreases. According to Laplace's law, this is paralleled by a decrease in the pressure. When a critical radius *R*_cyt_ ≈ 50 nm of the monolayers is reached, the rapid expansion of the cytosolic cap begins, overriding the rate of lateral TAG spreading. In parallel, the luminal cap is shrinking. In contrast to the simulated addition of PLs to a pre‐formed GUV‐pLD (cf. Fig. 3B), the shrinking of the luminal cap is not paralleled by a shrinking of the lateral TAG extension because the lateral pressure *p*_int_ (≤ 3 Pa, see dotted line in Fig. 4Diii) remains far below the effective pressure. Instead, the TAG disk in the inter‐monolayer space further expands, but at a low rate. The asymmetric cap growth is due to the small difference in surface tension between the cytosolic and luminal cap monolayer. During emptying of the luminal cap into the cytosolic cap, the surface tension of both cap monolayers decreases whereas the edge tensions change in opposite directions. The extension of the luminal cap amounts to only 30 nm at the time point of LD detachment, the fraction of residual TAG remaining in the bilayer and the luminal cap after detachment of the cytosolic cap is < 3%. The supply rate of TAG to the cytosolic cap monolayer corresponds strictly to the uptake rate into the pLD (see Fig. 4Di,iii and Eqn 20) and increases steeply in the second phase of pLD expansion. The supply rate of TAG per unit area of the cytosolic monolayer varies between 2 and 4 × 10^5^ min^−1^. The TAG density in the cytosolic monolayer rises in a few seconds from the equilibrium value of 6 × 10^4^ μm^−2^ to values of about 8 × 10^4^ μm^−2^, which remains below the maximal value of about 1 × 10^5^ μm^−2^ at 5% saturation. In the early phase of pLD expansion, the pressure reaches high values of about 50 kPa to fall continuously to about 10 kPa towards the end of the maturation. The steep increase of TAG loading in the second growth phase is paralleled by a steep increase of PLs taken up from the reservoir monolayer. At the time point of LD detachment, the amount of PLs additionally taken up from the reservoir monolayer amounts to about 6 × 10^5^ which is approximately 50% of the total PL content of the LD. The TAG/PL ratio of the newly formed LD is 21 × 10^6^/1.2 × 10^6^ ≈ 18, i.e. for the continuing formation of LDs of this size, PL *de novo* synthesis would have to be about 2% of the exported TAG to keep the total PL content of the ER membrane stable. The changes of the surface tension in the reservoir monolayers (≈ 0.03 mN·m^−1^) are much smaller than those of the cap monolayers (≈ 0.8–1.8 mN·m^−1^) due to the large size of the reservoir monolayer. The flow of PLs from the cytosolic reservoir monolayer into the expanding cytosolic cap monolayer increases the surface tension of the cytosolic reservoir monolayer faster than that of the luminal reservoir monolayer (Fig. 4Dx). If the delivery of PLs from the cytosolic monolayer to the cap monolayer is not immediately replenished by PL *de novo* synthesis, this asymmetric increase of the surface tension in the reservoir monolayers should increase the curvature of the ER bilayer.

**Fig. 5:**
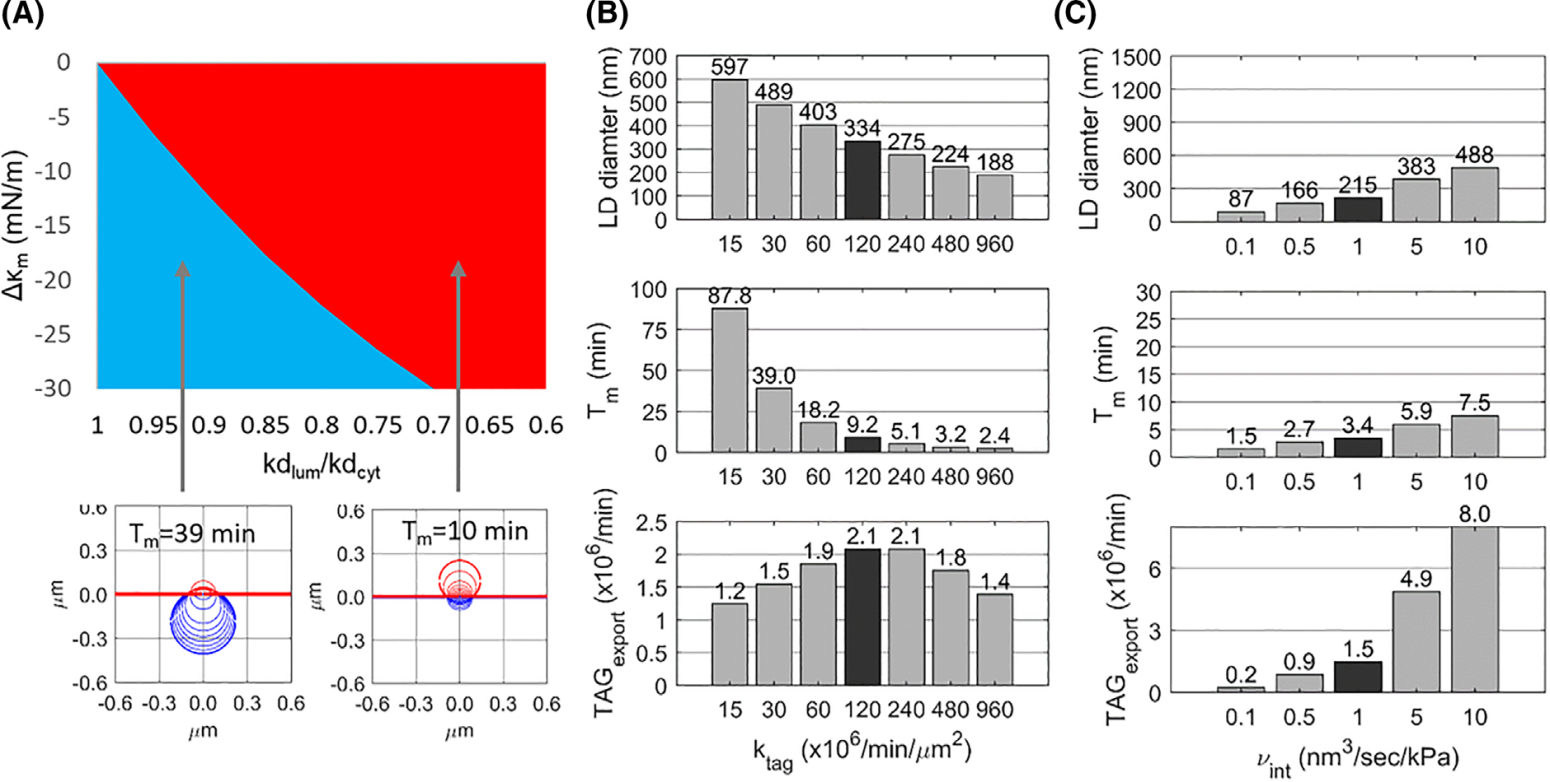
Impact of elastic and kinetic parameters on LD formation. (A) Simulated LD formation at the simultaneous variation of elastic moduli and kinetic properties of the monolayers. Elastic parameters varied, κ_m_(cyt) = 190 − Δκ_m_; κ_m_(lum) = 190 + Δκ_m_ with 0 < Δκ_m_ < 30 mN·m^−1^, the ratio kd_lum_/kd_cyt_ of kinetic parameters of PL flow varied between 0.6 and 1. The blue and red areas mark parameter combinations for which either a luminal or a cytosolic LD (depicted in the small panels below) is formed. Within these two ranges, the size and maturation time of either LD was virtually constant. (B) Impact of varying TAG uptake rates on size and maturation time of a cytosolic LD formed in a kinetically asymmetric bilayer with kd_lum_/kd_cyt_ = 0.5. (C) Impact of varying volume‐flow rates ν_int_ on size, maturation time, and TAG content of a cytosolic LD formed in a kinetically asymmetric bilayer with kd_lum_/kd_cyt_ = 0.5.

The simulations suggest that small asymmetries in the elastic or kinetic properties of the two monolayers may give rise to the formation of either a cytosolic or a luminal LD. These two types of monolayer asymmetry can act synergistically or antagonistically. Figure 5A illustrates how the directionality of LD formation is influenced by altering the elastic moduli κ_m_ of the two cap monolayers in favour of budding towards the lumen, while simultaneously reducing the PL flow rate in the luminal monolayer in favour of budding towards the cytosol. Depending on the extent of the mutually antagonising elastic and kinetic asymmetry, either a luminal or cytosolic LD is formed, indicated by the red and blue ranges in Fig. 5A. The cytosolic and luminal LD differ in size and maturation time. The luminal LD has more than twice the volume of the cytosolic LD. In addition, its maturation time is about four times longer (38 min versus 10 min). These differences occur because, at the preferred expansion of the luminal cap, the surface area of the cytosolic cap remains much smaller than in the opposite case. Hence, at a given fixed supply rate of TAG per unit area of the cytosolic monolayer, the solvation pressure and thus the flux of TAG into the pLD is lower than in the case of a predominantly expanding cytosolic monolayer.

The most remarkable result of the simulations shown in Fig. 5A is that despite a large shift of the elastic constants of the monolayers in favour of the formation of a luminal LD, a comparatively small reduction of the PL flow rate of about 50% in the luminal monolayer is sufficient to force the formation of a cytosolic LD. This finding suggests that the control of the direction of LD synthesis towards the cytosol or the lumen by selectively reducing the PL flow rate in one of the two monolayers is a mechanism that operates stably, independent of changes in the elastic parameters of the bilayer.

As shown in Fig. 5B, the rate of TAG supply to the cytosolic monolayer has a significant influence on the size and maturation time of the LD. Increasing rates of TAG supply result in smaller LDs, which are formed in a shorter period. A low TAG supply rate implies a low solvation pressure and thus a long first phase of pLD growth, characterised by a predominant lateral TAG flow in the bilayer before a critical cap radius is reached at which a notable expansion of the caps begins. This explains the larger size of LDs and the prolonged maturation time at low TAG synthesis rates. Remarkably, the concomitant changes in size and maturation time have little effect on the TAG export rate, i.e. the amount of TAG delivered to the cytosolic LD pool by a single LD per time unit. In the chosen example, the maximal TAG export rate is achieved with TAG supply rates of *k*_tag_ ≈ 1 × 10^8^ min^−1^·μm^−2^. An eight‐fold lower rate of TAG supply results in a maturation time of more than 1 h, but the TAG export rate is still about 50% of the maximum.

A third model parameter influencing the outcome of simulated LD formation is the lateral volume‐flow rate $\nu_{\mathrm{int}}$ of TAG within the bulk bilayer (see Fig. 5C). The value of this parameter depends on the chemical composition of the bilayer and the presence of membrane proteins interfering with the lateral mobility of the condensed pLD (see Discussion). Decreasing or increasing the value of $\nu_{\mathrm{int}}$ by one order of magnitude concerning the standard value generates LDs with diameters between 200 and 650 nm. Since the LD volume of a spherical LD scales with the cube of the diameter, the range of volume changes is approximately a factor of 35.

## Discussion

This work proposes a biophysical‐biochemical model of LD formation, which integrates the lateral flow of PLs and related density changes into the elementary biophysics of membrane elasticity. This extension of the ‘classical’ elasto‐mechanical description of membrane deformations was necessary because, without the additional supply of PLs, an expansion of more than 2–3% of the pLD‐covering PL is hardly conceivable. Essentially, the proposed model postulates that the accumulation of TAG in the cytosolic monolayer of the ER membrane generates a solvation pressure that forces TAG into the interface between the two monolayers, where it condenses into an incompressible oil drop. In the first phase of growth, this oil drop forms a laterally elongated lens due to the large bending energy of the monolayers, which prevents significant expansion towards the cytosol or lumen. As the curvature of the cap, and thus the bending energy, fall below critical values, the solvation pressure becomes sufficient to drive the expansion of the spherical caps up to contact angles at which the edge tension of the pLD neck exceeds the rupture tension and detachment of the cap in the sense that an LD occurs.

To demonstrate the feasibility of the model and to constrain the numerical values of some model parameters, simulations were performed recapitulating experimental results obtained with synthetic pLDs embedded in a bilayer of GUVs. Changing the surface tension of the cytosolic monolayer by mechanical forces or by incorporating additional PLs shifted the initially fully symmetric pLD in the direction of the applied tension gradient (see Fig. 3). These results are consistent with numerous experiments showing that asymmetry between cytosolic and luminal monolayer surface tensions imposes spatial directionality on LD formation [5, 41].

Application of the model to *in vivo* formation of LDs provided several interesting insights.

### Additional PLs are required to allow monolayer expansions in the μm range

Due to the high area expansion modulus of 200–250 mN·m^−1^ of PL monolayers, bulging of the monolayer of several hundred nanometres can only be achieved by recruiting additional PLs to the surface of the bulge (see Fig. 2C). This may be accomplished by *de novo* synthesis of membrane lipids, smoothing of monolayer undulations, and lateral influx of PL into the region with locally increased surface tension. It has been observed that LDs are preferentially synthesised at ER regions with high curvature, e.g. tubules [42]. A high degree of curvature of the bilayer reduces the bending stiffness of the cap monolayer in the early growth phase of the pLD. The continuous re‐synthesis of PL is obligatory for a membrane that constantly forms LDs and releases a plethora of different vesicles to other membranes of the cell. Compared to TAG synthesis, only a few percent of total FA esterification at the ER has to be directed to PL synthesis to replenish the PL loss due to LD synthesis (cf. Fig. 4Dvi,vii). It is conceivable that this replenishment can be achieved by PL synthesis localised to the surface of the expanding cytosolic cap, i.e. without the contribution of PL influx from the reservoir monolayer.

### Low mobility of pLD in inter‐monolayer space is essential

The lateral expansion rate of the pLD within the inter‐monolayer space ultimately determines the size of the LD. To produce realistic LDs of 200–500 nm in size within a maturation time of 5–15 min, the rate constant for the lateral TAG flow had to be put to values of ν_int_ ≈ 0.1–1 nm^3^·s^−1^·kPa^−1^. In simulations with a volume flow rate of ν_int_ = 1 nm^3^·s^−1^·kPa^−1^, the front of the inter‐monolayer TAG disk had just travelled about $x_{\mathrm{int}}\approx 100\;\mathrm{nm}$ away from the pLD centre. Equating this distance with the mean square displacement <*x* ^2^ > = 2*Dt* of freely diffusing TAG molecules gives a diffusion coefficient of *D* ≈ 10^−5^ μm^2^·s^−1^ that is about five orders of magnitude smaller than the diffusion coefficients *D* ≈ 4 μm^2^·s^−1^ reported for freely diffusing TAG molecules [43]. Thus, it appears that nucleated TAG of the pLD behaves like a lipid domain with extremely low mobility within the inter‐monolayer space. It remains unclear, whether the TAG domain is virtually immobile *per se*, or whether integral membrane proteins resident at the interface between pLD and the bilayer slow down the lateral TAG flow by acting as a kinetic barrier. Kuzmin *et al*. [44] predicted that relatively small changes in the height of such kinetic barriers can dramatically alter the times for domain mobility in free‐standing bilayers by as much as 0.1 to 10^10^ s.

### Small bilayer asymmetries decide on the directionality of LD budding

A marginal asymmetry between the elastic and/or kinetic properties of the cytosolic and luminal monolayer of the ER membrane is sufficient to drive the expansion of the pLD into the direction of the cytosol or lumen. In the perfectly symmetric bilayer, the caps of the developing pLD are in an unstable quasi‐equilibrium with the chance of one cap absorbing the other. To control the directionality of LD formation towards the cytosol or lumen, the so‐called Ostwald ripening is forced by generating a difference between the surface tensions of the two monolayers. In some cell types (e.g. hepatocytes, enterocytes), LDs can also be released into the lumen of the ER in the form of lipoproteins. This fact makes it likely that the directionality of detachment is not essentially dependent on the structural asymmetry of the ER bilayer *per se*, but on the dynamics with which alterations of the PL composition and recruitment of additional membrane proteins can be managed. Choudhary *et al*. [45] have pointed out that the intrinsic molecular curvatures of ER PLs can already determine whether LDs remain embedded in or emerge from the ER. On top, membrane proteins may change the elastic and kinetic properties of PL monolayers in several ways: formation of non‐covalent bonds between amino acid residues and membrane lipids [41, 46] or catalysis of chemical modifications of PLs [45], both affecting the inter‐molecular elastic force constants of membrane lipids. Integral membrane proteins may also change the PL mobility of the monolayers by acting as steric obstacles [47] and inducers of lipid domains with largely differing PL mobility [48, 49]. The simulation results summarised in Fig. 5 suggest that diminishing the rate of the PL flux in the luminal monolayer represents an effective means to enforce the directionality of pLD expansion towards the cytosol even in cases where the elastic moduli of the monolayers would favour an expansion into the opposite direction. Given the large number of proteins now reported to support pLD development in the ER bilayer (reviewed e.g. by Zadoorian *et al*. [50]) with each of them affecting the elastic moduli of the monolayers in a specific way, reducing the PL flux by just one distinct protein would be sufficient to robustly control the direction of LD synthesis.

The fact that the microsomal transfer protein, which is essential for the formation of luminal very low‐density lipoprotein (VLDL), possesses PL transfer activity [51] suggests that the selective increase of PL supply to one of the two monolayers may be crucial for the direction of LD synthesis. Even if the transfer activity for TAG is absent, as in *Drosophila*, VLDL synthesis is possible [52]. It is well conceivable that during the synthesis of VLDL, PLs are transferred by the microsomal transfer protein from the cytosolic monolayer to the luminal monolayer to keep its surface tension relatively lower.

**Fig. 6:**
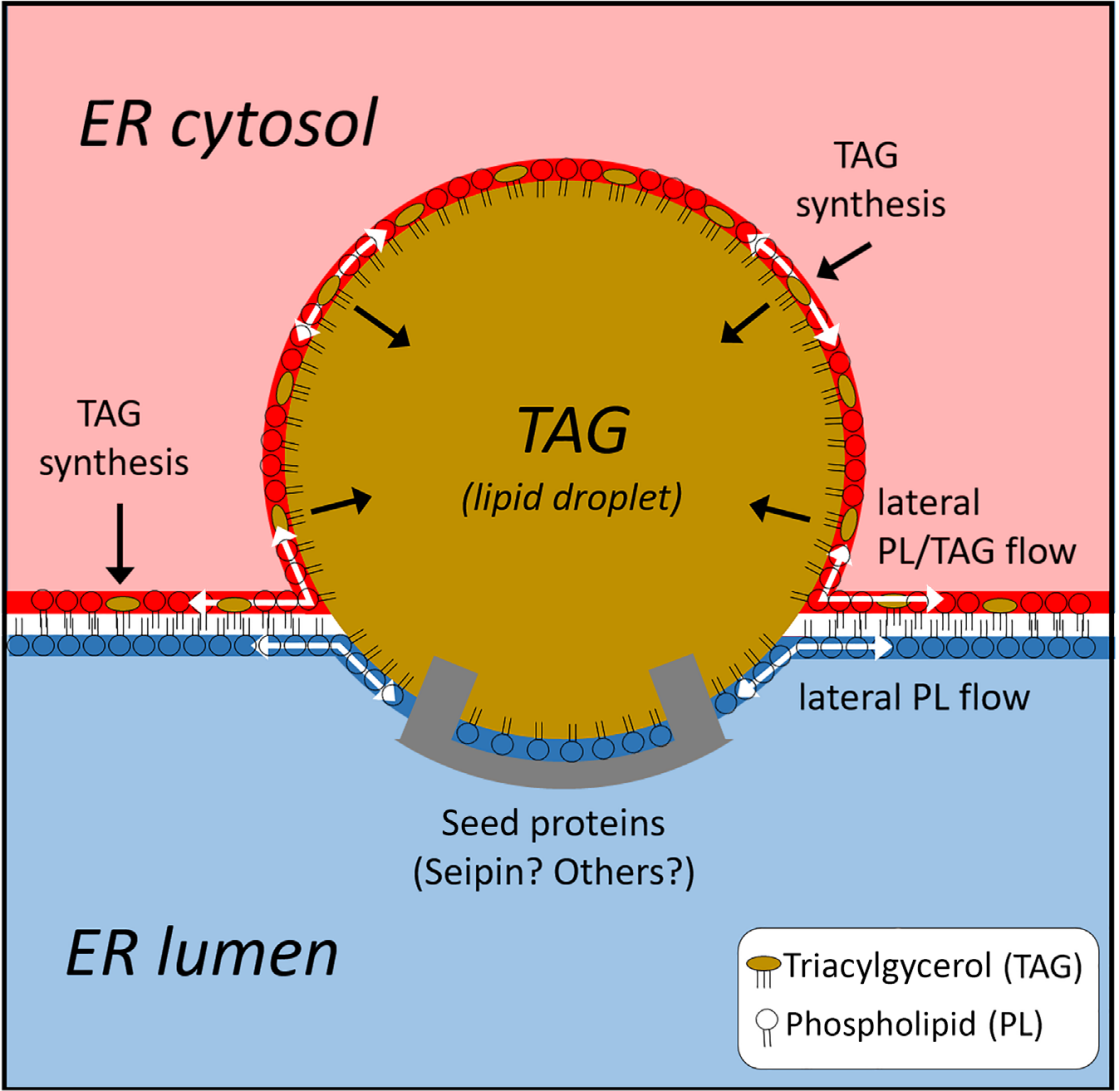
Working hypothesis for LD formation at the ER membrane. The site of LD formation is defined by the attachment of specific membrane proteins, including the luminal protein seipin and possibly other proteins. They may have three functions: Acceleration of TAG nucleation, local fixation of the pLD by reducing lateral mobility, and relative increase in surface tension of the luminal cap monolayer by increasing its elasto‐mechanical rigidity, and/or reducing the influx of phospholipids from the adjacent reservoir monolayer. TAG is newly synthesised in the cytosolic monolayer, but can also flow into the cap monolayer from the adjacent reservoir monolayers due to its high lateral mobility. The accumulation of TAG in the cap monolayers creates a solvation pressure that causes the lateral and transversal expansion of the pLD. The cytosolic cap of the pLD adopts a budding conformation and is finally separated from the surrounding monolayer as a new LD when the edge tension between the cap monolayer and the reservoir monolayer exceeds a critical threshold. Kim *et al*. [24] arrived at a similar scheme when summarising the results of MD simulations of LD formation.

It is tempting to speculate that the two conditions for the formation of LDs with reasonable size – a slow lateral volume flow of TAG in the inter‐monolayer space and a relatively higher surface tension of the luminal cap monolayer – are caused by the same molecular mechanism (see Fig. 6). A possible candidate protein is seipin [53]. The transmembrane segments of this luminal protein have been shown to have a critical function in TAG nucleation [54]. In *Saccharomyces cerevisiae*, seipin assembles into a decameric complex with the shape of a dome‐shaped cage, where the luminal domains form a stable ring at the bottom of the cage and the transmembrane segments form the sides and top of the cage [55]. It is possible that the hydrophobic luminal domains of seipin hinder the lateral mobility of the nucleated TAG domain and thus prevent the formation of larger LDs. It is well known that transmembrane proteins can significantly reduce the mobility of lipid domains [47], even when the proteins are much smaller than the domain itself [56]. The idea that seipin inhibits lateral spread of TAG is consistent with the observation that seipin deficiency may lead to the formation of larger LDs [15]. A second function of the hydrophobic domains of seipin may be to reduce the rate of lateral PL flow within the luminal monolayer. This idea is supported by the observation that human seipin binds to anionic PLs [57]. Evolutionarily, the ability of cells to synthesise energy in the form of polymeric carbohydrates and FA esters may have been a crucial selective advantage established early in biological evolution. One can imagine a simple mechanism of LD formation in primitive cells, operating without the assistance of various regulatory proteins: TAG and PL are constantly synthesised at the cytosolic monolayer. At high concentrations in the monolayer, TAG accumulates in the inter‐monolayer space where it can spontaneously and randomly nucleate. At the surface of the forming pLD, PL mobility is naturally reduced [58], favouring a rapid increase of surface tension, but only in the luminal monolayer as newly synthesised PLs are preferentially added to the cytosolic monolayer. The resulting asymmetry in surface tension drives the expansion of the pLD towards the cytosol.

### TAG supply determines LD size and maturation time, but has little effect on filling efficiency

The model simulations suggest that the rate of TAG synthesis has a significant impact on the size and maturation time of LDs. However, when the maximum rate of TAG synthesis (ku_tag_) was varied by two orders of magnitude, the simulations only yielded variations by a factor of two for the TAG export rate per LD, meaning that smaller size and shorter formation time almost cancel each other out. This finding implies that cells challenged by a high free FA load have no other choice than to increase the number of LD formation sites on the ER membrane. It is still largely unknown how the synthesis of TAG at the ER membrane and the formation of LDs are coordinated. Listenberger *et al*. [59] reported, that the incubation of CHO cells with free FAs increases their esterification to TAG. They found that the esterification of palmitate was strongly stimulated by oleic acid, thus reducing the cellular level of this potentially lipotoxic FA. Oleate has been described as a strong stimulator of LD synthesis, promoting the initiation of pLD formation sites at the ER. [60]. Borradaile *et al*. [61] reported that the rapid incorporation of palmitate into saturated PL and TAG species in microsomal membranes of CHO cells was associated with a dramatic dilatation of the ER. This finding suggests that the deposition of TAG within the ER membrane may exceed the LD‐dependent TAG export into the cytosol, i.e. that there is no efficient negative feedback between LD formation and TAG synthesis.

In the model, it was assumed that all TAG in pLDs originates from the monolayer of the cytosolic cap where it is either synthesised directly or recruited from other regions of the ER membrane by lateral transport. However, there are two forms of the enzyme diglyceride acyltransferase (DGAT), which catalyses the final step of TAG synthesis. DGAT1 is proposed to contribute to TAG synthesis on both sides of the ER [62]. Moreover, MD simulations have provided evidence that TAG transits much faster from the cytosolic to the luminal monolayer compared with the slow flip‐flop of PLs [4]. Thus, the possibility that TAG resident in the luminal monolayer also feeds the formation of LDs cannot be ruled out. How the synthesis of TAG is kinetically adapted to the uptake rate in LDs is not yet known. In the model, this adaptation was achieved phenomenologically by making TAG uptake into the cytosolic monolayer proportional to the difference between current and maximal membrane concentration.

At this point, it should be noted that the lipid composition of the pLD also has a significant influence on the size and rate of LD formation. For example, if TAG and a cholesteryl palmitate ester (CPE) are synthesised in the cap monolayer and transferred into the pLD in a 1 : 1 ratio, then, in comparison with the development of the TAG‐LD shown in Fig. 4D, the formation time and volume of the TAG/CPE‐LD would increase by a factor of about 1.5 from 10 to 16 min and 0.02 to 0.03 μm^3^. This is due to the higher density of nucleated CPE (ρ_CPE_ = 1.066 g·cm^−3^ [63] versus ρ_TAG_ = 0.91 g·cm^−3^), which requires a higher minimum concentration of CPE in the cap monolayer (8.4 × 10^8^ μm^−3^ versus 6.2 × 10^8^ μm^−3^) for the generation of an inward pressure.

### Limitations of the modelling approach

The mathematical model presented in this work is a generic one aiming at shedding some light on the principles governing the growth of lipid aggregates in the ER bilayer. Many molecular details of LD genesis, in particular those related to the specific function of membrane proteins that bind to the cytosolic and luminal surface of the growing LD, have not been included in the model. It is therefore very likely that the elastic and kinetic model parameters used are not static, but may change during the development of the LD. Below are some facts that have not been considered in the model or that may affect the values of model parameters. (a) LDs embedded in a model cell membrane form a PL diffusion barrier [58], i.e. the exchange rate of PLs between the cap and reservoir monolayer may be an order of magnitude lower than that measured in pure PL bilayers. As mentioned earlier, this fact alone, together with an ongoing PL supply exclusively to the cytosolic monolayer, would be sufficient to drive pLD expansion into the cytosol. (b) LDAF1 is a transmembrane protein that initially forms an oligomeric complex with the luminal transmembrane protein seipin and later diffuses into the cytoplasmic monolayer during pLD expansion, where it facilitates the formation of LDs at low rates of TAG synthesis [16]. In light of the model simulations, this detachment‐promoting effect of LDAF1 could be due to its ability to lower the elastic moduli of the cytosolic monolayer, thus allowing expansion at lower solvation pressures. (c) In the model, TAG enters the pLD only from the monolayer covering the cytosolic cap. One question that has not yet been resolved concerns the capacity of TAG and PL synthesis at the cytosolic cap monolayer. There are two alternative possibilities: With the recruitment of initialization proteins, a fixed synthesis capacity for TAG and PL is allocated, independent of the size of the pLD. Alternatively, the allocation of the synthesis capacity for TAG and PL to the cytosolic monolayer rises proportional to the surface area of the cap, as assumed in the model. Moreover, experiments by Salo *et al*. [64] suggest that TAG may flow from one pLD to the neighbouring one, and this transit is facilitated by seipin. Such lateral transfer of TAG from one pLD to the other would prevent pLDs equipped with insufficient protein machinery required for budding from growing steadily but collapsing into perfectly growing pLDs. (d) A fundamental question concerns the energy supply that drives the expansion of the pLD. The proposed model postulates the existence of a solvation energy that is related to the free energy released when TAG passes from the monolayer into the inter‐monolayer space. However, an additional energetic contribution may originate from the strongly exergonic esterification of the activated acyl‐CoA moieties with the glycerol backbone. Whether and by which molecular mechanism this chemical energy can be converted into a pressure‐driving pLD expansion remains to be clarified. Finally, a third energetic contribution may result from TAG nucleation, which represents an exergonic process [65, 66]. The capture of TAG molecules, which initially move rapidly in the bilayer, into the already existing TAG nucleus is associated with a reduction of the free energy. This free energy release may be equivalent to a certain ‘nucleation pressure’, similar to the transition of TAG from the monolayer into the pLD.

## Methods

### Simulation protocol

All *ab initio* simulations started with a small volume of a very flat pLD defined by the cap coordinates $x_{\mathrm{cyt}}=x_{\mathrm{lum}}=0.001\;\mathrm{nm}$ and the lateral extension *x*_int_ = 7 nm corresponding to the diameter of the hexagonal ring formed by the membrane protein seipin, which has been described as a central seed protein for TAG nucleation during the initial phase of pLD formation [26, 27, 28]. All numerical simulations were carried out using the programming platform matlab [67].

## Conflict of interest

The author declares no conflict of interest.

## Data Availability

Data sharing is not applicable to this article as no new data were created or analysed in this study.
